# XA21-specific induction of stress-related genes following *Xanthomonas* infection of detached rice leaves

**DOI:** 10.7717/peerj.2446

**Published:** 2016-09-28

**Authors:** Nicholas C. Thomas, Benjamin Schwessinger, Furong Liu, Huamin Chen, Tong Wei, Yen P. Nguyen, Isaac W.F. Shaker, Pamela C. Ronald

**Affiliations:** 1 Department of Plant Pathology and the Genome Center, University of California, Davis, CA, United States; 2 Joint BioEnergy Institute, Emeryville, CA, United States; 3 Research School of Biology, Australian National University, Acton, Australia; 4 State Key Laboratory for Biology of Plant Diseases and Insect Pests, Institute of Plant Protection, Chinese Academy of Agricultural Sciences, Bejing, China

**Keywords:** XA21, *Xanthomonas*, EFR, Rice, RNAseq

## Abstract

The rice XA21 receptor kinase confers robust resistance to the bacterial pathogen *Xanthomonas oryzae*pv. *oryzae* (*Xoo*). We developed a detached leaf infection assay to quickly and reliably measure activation of the XA21-mediated immune response using genetic markers. We used RNA sequencing of elf18 treated EFR:XA21:GFP plants to identify candidate genes that could serve as markers for XA21 activation. From this analysis, we identified eight genes that are up-regulated in both in elf18 treated EFR:XA21:GFP rice leaves and *Xoo* infected XA21 rice leaves. These results provide a rapid and reliable method to assess bacterial-rice interactions.

## Introduction

Plant immunity is mediated, in part, by cell surface immune receptors that recognize molecules produced by microbes. For example, the *Arabidopsis* FLS2 (Flagellin Sensing 2) and EFR (Elongation Factor Tu Receptor) receptors recognize the flg22 peptide derived from bacterial flagellin and the elf18 peptide derived from elongation factor thermo-unstable (EF-Tu) protein, respectively (Gomez-Gomez & Boller, 2000; Zipfel et al., 2006). The rice XA21 receptor recognizes the sulfated RaxX peptide (RaxX21-sY) derived from the RaxX protein produced by *Xanthomonas oryzae* pv. *oryzae* (*Xoo*) (Song et al., 1995; Pruitt et al., 2015; Wei et al., 2016). XA21, EFR, and FLS2 all contain extracellular leucine rich repeat (LRR), transmembrane, and intracellular non-RD (arginine-aspartic acid) kinase domains. These receptor domains are partially interchangeable. For example, the LRR domain from EFR can be fused to the transmembrane and intracellular domain of FLS2 to form a chimeric receptor that responds to elf18 treatments when transiently expressed in *Nicotiana benthamiana* and *Arabidopsis thaliana* (Albert et al., 2010). The EFR LRR can be fused to the transmembrane and intracellular domain of XA21 to form a chimeric receptor that responds to elf18 treatment and confers partial resistance to *Xoo* in transgenic rice lines (Schwessinger et al., 2015a).

The availability of rapid and reliable assays that measure markers characteristic of immune response activation can help facilitate investigations of innate immune signaling. For example, immune signaling studies of FLS2 and EFR in *Arabidopsis* have been aided by the availability of rapid and reliable assays (Gomez-Gomez & Boller, 2000; Zipfel et al., 2006; Chinchilla et al., 2007; Lu et al., 2010; Albert et al., 2010; Schulze et al., 2010; Schwessinger et al., 2011; Sun et al., 2013; Li et al., 2014). In contrast, studies of the XA21-mediated immune response have been limited by the lack of rapid assays and well-characterized genetic markers. Typically, disease assessments are carried out by measuring lesions on rice leaves or by assessing bacterial populations from infected leaves (Kauffman et al., 1973; Song et al., 1995; Da Silva et al., 2004; Park et al., 2008; Chen et al., 2014; Pruitt et al., 2015).

In this study we aimed to establish a rapid and efficient assay to monitor the XA21-mediated immune response after bacterial infection. For this purpose, we employed the EFR:XA21:GFP chimera composed of the EFR extracellular domain and the XA21 transmembrane and intracellular kinase domains, tagged with green fluorescent protein (EFR:XA21:GFP) (Schwessinger et al., 2015a). EFR:XA21:GFP transgenic rice plants are partially resistant to *Xoo* and detached EFR:XA21:GFP leaves respond to elf18 with stress-related gene induction, mitogen-activated protein kinase (MAPK) cascade activation, and reactive oxygen species (ROS) production (Schwessinger et al., 2015a). These results indicate that plants expressing the EFR:XA21:GFP chimeric protein are appropriate for studies to identify markers of resistance.

We used RNA sequencing (RNAseq) to identify genes differentially regulated in elf18 treated EFR:XA21:GFP rice. We then assessed if differentially regulated genes (DRGs) in elf18 treated EFR:XA21:GFP rice leaves were up-regulated in *Xoo* infected rice leaves expressing full-length XA21, which are resistant to *Xoo*. We developed a rapid and reliable assay to analyze gene expression in detached rice leaves inoculated with *Xoo*. We identified 8 DRGs from elf18 treated EFR:XA21:GFP rice that are also specifically up-regulated in detached XA21 rice leaves infected with *Xoo*.

## Materials and Methods

### Plant growth, peptide and bacterial treatments of detached rice leaves

For peptide treatments, wild type (WT) Kitaake and progeny from line EFR:XA21:GFP-3-8 (EFR residues 1-649 and XA21 residues 651-1025 called EFR:XA21:GFP in this study) Kitaake rice leaves were harvested from plants grown in the greenhouse for 4.5 weeks (Schwessinger et al., 2015a). 1.5–2 cm leaf sections were collected from expanded adult leaves using surgical grade scissors. Tissue from the leaf base and leaf tip was discarded. Detached leaves were equilibrated overnight in 6-well Costar cell culture plates under constitutive light (between 5–10 µmol/(m^2^*s)) (Fig. S1). Peptide treatments were performed in the morning and collected at the indicated times.

For bacterial inoculations, we used detached rice leaves harvested from 4-week old plants grown using a hydroponic growth system as described previously (Pruitt et al., 2015) under a light intensity of 280 µmol/(m^2^*s). Freshly harvested leaves from Kitaake and Ubi-Myc:XA21 Kitaake rice (called Myc:XA21 rice in this study) (Park et al., 2010) were cut into 1.5–2 cm pieces and immediately (without overnight equilibration) floated on 10 mM MgCl_2_ solution for mock treatments or 10 mM MgCl_2_ containing fresh *Xoo* cell suspensions at O.D._600_ of 0.1 (approximately 1 × 10^7^ cells mL^−1^). The samples were left overnight under constitutive light (between 5–10 µmol/(m^2^*s)) and collected 24 h post-inoculation (hpi). Leaves were floated on approximately 1.5 mL *Xoo* cell suspension media in 6-well Corning Costar cell culture plates (Fig. S1). The detached leaf infection assay allows a more uniform distribution, compared to the scissor inoculation method (Kauffman et al., 1973), of *Xoo* inoculum by floating leaves on bacterial suspensions.

### RNA isolation and qPCR gene expression analysis for peptide treated and bacterial infected leaf samples

Detached leaves were frozen in liquid nitrogen and powdered using a Qiagen tissuelyser. For tissue from greenhouse grown plants, RNA was extracted from powdered tissue using TRI Reagent and precipitated with isopropanol. For tissue from hydroponically grown plants, RNA was extracted using the Spectrum Plant Total RNA Kit from Sigma-Aldrich. RNA was DNase treated using the TURBO DNase kit from Life Technologies. RNA concentrations were normalized to the lowest sample concentration in each experiment. cDNA was synthesized from 2 µg of total RNA using the High Capacity cDNA Reverse Transcription Kit by Life Technologies. Gene expression changes were determined by ΔΔCt method (Schmittgen & Livak, 2008) normalized to *Actin* (*LOC_Os03g50885*) and compared to mock treated samples.

### Identification of genes differentially regulated in elf18 treated EFR:XA21:GFP rice using RNA sequencing

Plant growth, leaf tissue isolation, and treatments were performed as described above. RNA was isolated from untreated Kitaake as well as untreated and elf18 treated EFR:XA21:GFP leaf tissue using the Spectrum Plant Total RNA Kit from Sigma-Aldrich and on-column DNase treated to remove genomic DNA contamination following the manufacturer’s instructions. RNA was quantified using the Quant-IT Ribogreen RNA Assay Kit. Sequences were deposited to the NCBI Sequence Read Archive (BioProject ID PRJNA250865).

RNAseq libraries, sequencing, and reference alignment were performed as described previously (Schwessinger et al., 2015a). Sample correlation between Kitaake and EFR:XA21:GFP replicates and differential gene expression analysis was performed using the Bioconductor ‘edgeR’ package for R (Robinson, McCarthy & Smyth, 2010; McCarthy, Chen & Smyth, 2012).

### Bacterial strains and generation of mutants

We generated a PXO99AΔ*hrpA1* mutant in Philippine race 6 strain PXO99Az, a derivative of strain PXO99 (referred to as PXO99A in this study) (Salzberg et al., 2008). *Xoo* was grown in 10 g PSB (10 g Peptone (bacto-Peptone), 10 g Sucrose, 1 g sodium glutamate (glutamic acid, monosodium salt), final volume 1L, pH 7.0) or on PSA plates (PSB with 16 g/L bacto-agar) at 28°C. PXO99AΔ*hrpA1* was generated by single crossover mutagenesis using the suicide vector pJP5603 (Penfold & Pemberton, 1992). An approximately 500 base pair sequences within *hrpA1* was amplified using forward primer 5′-CGGGGTACCGTGCTGCGTGATTTGTCCG-3′and reverse primer 5′-CGCGGATCCTGACTTGGTCGATGCAGTCC-3′and cloned into the multiple cloning site of pJP5603 using the restriction enzyme sites KpnI and BamHI. PXO99A-competent cells were transformed with the suicide plasmids by electroporation and plated to PSA with kanamycin (50 µg/ml). PXO99AΔ*hrpA1* colonies with kanamycin resistance were screened by PCR for colonies with single crossover events, which contain the vector disrupting the target gene. PXO99AΔ*raxST* and PXO99AΔ*raxST(raxST)* complemented strains used in this study were described previously (Pruitt et al., 2015). PXO99AΔ*raxST* evades XA21-mediated immunity while the complemented PXO99AΔ*raxST(raxST)* strain does not.

## Results

### RNAseq analysis identifies 2212 genes that are differentially regulated in elf18 treated EFR:XA21:GFP rice leaves

**Figure 1:**
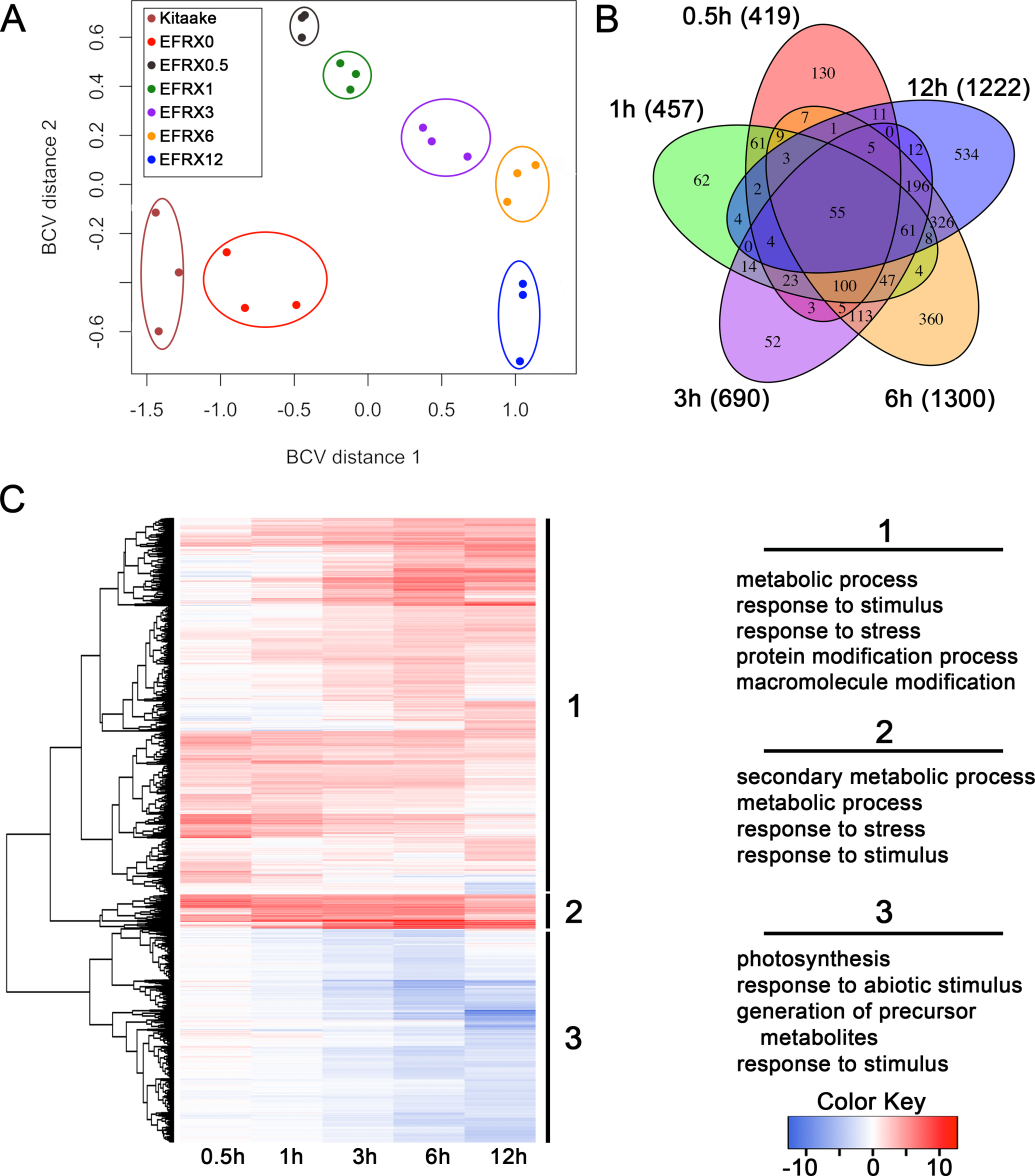
The transcriptomic profile of elf18 treated EFR:XA21:GFP rice is enriched for stress response related and photosynthesis-related genes. (A) Multi-dimensional scaling comparing biological coefficients of variance between each sample. Samples labeled Kit0 are Kitaake rice leaf samples without treatment. Samples labeled Kitaake represent untreated Kitaake samples at 0 h, EFRX represent EFR:XA21:GFP untreated samples (EFRX0) and samples treated with 500 nM elf18 at 0.5 h (EFRX0.5), 1 h (EFRX1), 3 h (EFRX3), 6 h (EFRX6), and 12 h (EFRX12). Groups of technical replicates are circled and sample color codes are indicated in upper left legend. (B) A five-way Venn diagram indicating number of total (indicated in parentheses), unique and overlapping differentially regulated genes between time points. (C) Heatmap representing expression levels of differentially regulated genes (DRGs) for EFR:XA21:GFP samples treated with elf18 for indicated durations. The three major DRG clades, determined by expression profile, are labeled 1, 2 and 3 and are indicated to the right of the heatmap. Significantly enriched gene ontology terms with a false discovery rate less than 0.5, compared to the reference, for each clade are shown on the right under the respective clade number. The heatmap color key indicates log_2_ fold change values compared with untreated, EFR:XA21:GFP 0 h samples.

We analyzed the transcriptomic profile of EFR:XA21:GFP rice lines treated with elf18 to identify genes differentially regulated during this response. We sequenced cDNA from EFR:XA21:GFP leaves treated with 500 nM elf18 for 0.5, 1, 3, 6, and 12 h. We also included untreated EFR:XA21:GFP and Kitaake as controls (Table S1). Multidimensional scaling of pairwise biological coefficient of variance comparisons for each sample revealed that replicate samples group together (Fig. 1A). This grouping of biological replicates demonstrates the overall transcriptional similarity between each sample (Robinson, McCarthy & Smyth, 2010).

We identified 2,212 genes that were differentially regulated in EFR:XA21:GFP rice treated with elf18 compared with untreated (0 h) samples. Using a false discovery rate (FDR) cutoff of 0.05 and absolute expression log fold change (logFC) of 2 or greater, we previously reported that the transcriptomic profile of untreated Kitaake compared to untreated EFR:XA21:GFP did not differ significantly (Schwessinger et al., 2015a). Over the treatment time course, we identified 2,212 DRGs (FDR <0.05, absolute logFC >2) using untreated EFR:XA21:GFP at 0 h as a reference. The number of DRGs that overlap between the elf18 treatment time points are summarized in Fig. 1B and File S1. Of the 2,212 differentially regulated genes, there were 1,420 up-regulated and 792 down-regulated genes. The highest number of DRGs (1,504) was observed 6 h post elf18 treatment. These results show that elf18 treated EFR:XA21:GFP rice express a substantially different set of genes over time compared to untreated (0 h) samples.

### Stress response related genes are up-regulated while photosynthesis related genes are down-regulated in EFR:XA21:GFP rice treated with elf18

To examine the types of biological processes affected in elf18 treated EFR:XA21:GFP rice, we analyzed GO term enrichment of DRGs using the AgriGo analysis tool (Du et al., 2010). A total of 1,204 out of 1,420 of the up-regulated DRGs and 682 of the 806 down-regulated DRGs had GO annotations. An FDR of 0.05 or less was used to define significantly enriched terms compared to the Michigan State University annotation reference as calculated by the AgriGo tool (Du et al., 2010; Kawahara et al., 2013). Fig. 1C and File S2 summarize the most enriched GO terms in each of the three major DRG clades. Clade 1 contains 1,333 genes that are mostly up-regulated over time. Genes from clade 1 are enriched for metabolic process (GO:0008152), response to stimulus (GO:0050896) and response to stress (GO:0006950) GO terms (Fig. 1C). Clade 2 genes (122) are up-regulated across all time points and are enriched for secondary metabolic process (GO:0019748), metabolic process (GO:0008152) and response to stress (GO:0006950) GO terms (Fig. 1C). Clade 3 consists of 757 genes that are mostly down-regulated in all timepoints. Photosynthesis (GO:0015979) and response to abiotic stimulus (GO:0009628) are the most enriched GO terms associated with clade 3 genes (Fig. 1C).

### qPCR validation of genes up-regulated in elf18 treated EFR:XA21:GFP plants

We chose 23 DRGs from the elf18 treated EFR:XA21:GFP rice RNAseq dataset with relatively high logFC and low FDR values after 3, 6, and 12 h for detailed analysis. We first assessed if the 23 genes up-regulated in elf18 treated EFR:XA21:GFP could be validated by qPCR analysis. Eleven out of 23 DRGs were up-regulated in EFR:XA21:GFP rice leaves after elf18 treatment. Transcripts of the remaining 12 candidate genes were detectable by qPCR amplification but were not up-regulated in elf18 treated EFR:XA21:GFP leaves (File S3).

### Establishment of bacterial infection assay of detached rice leaves

**Figure 2:**
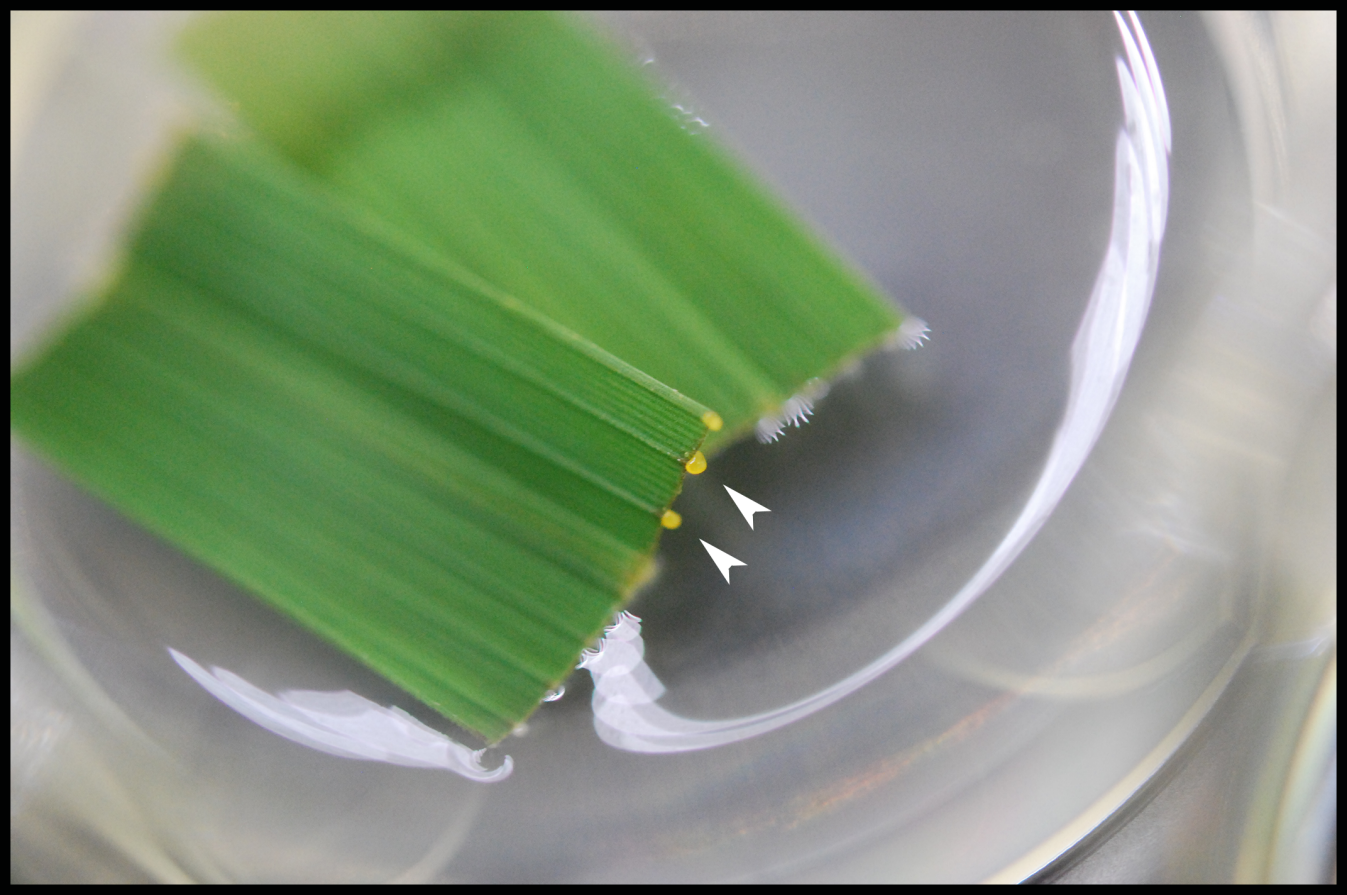
Bacterial oozes from an infected rice leaf. Bacterial oozing (white arrowheads) was observed from rice leaf xylem vessels three days post infection. This image shows detached Kitaake rice leaves infected with PXO99A in a 6-well cell culture plate. Bacterial oozing was consistently observed in Kitaake and Myc:XA21 detached leaves infected with PXO99A. Rice leaves were collected from 4-week old, hydroponically grown plants and floated on *Xoo* cell suspension media.

**Figure 3:**
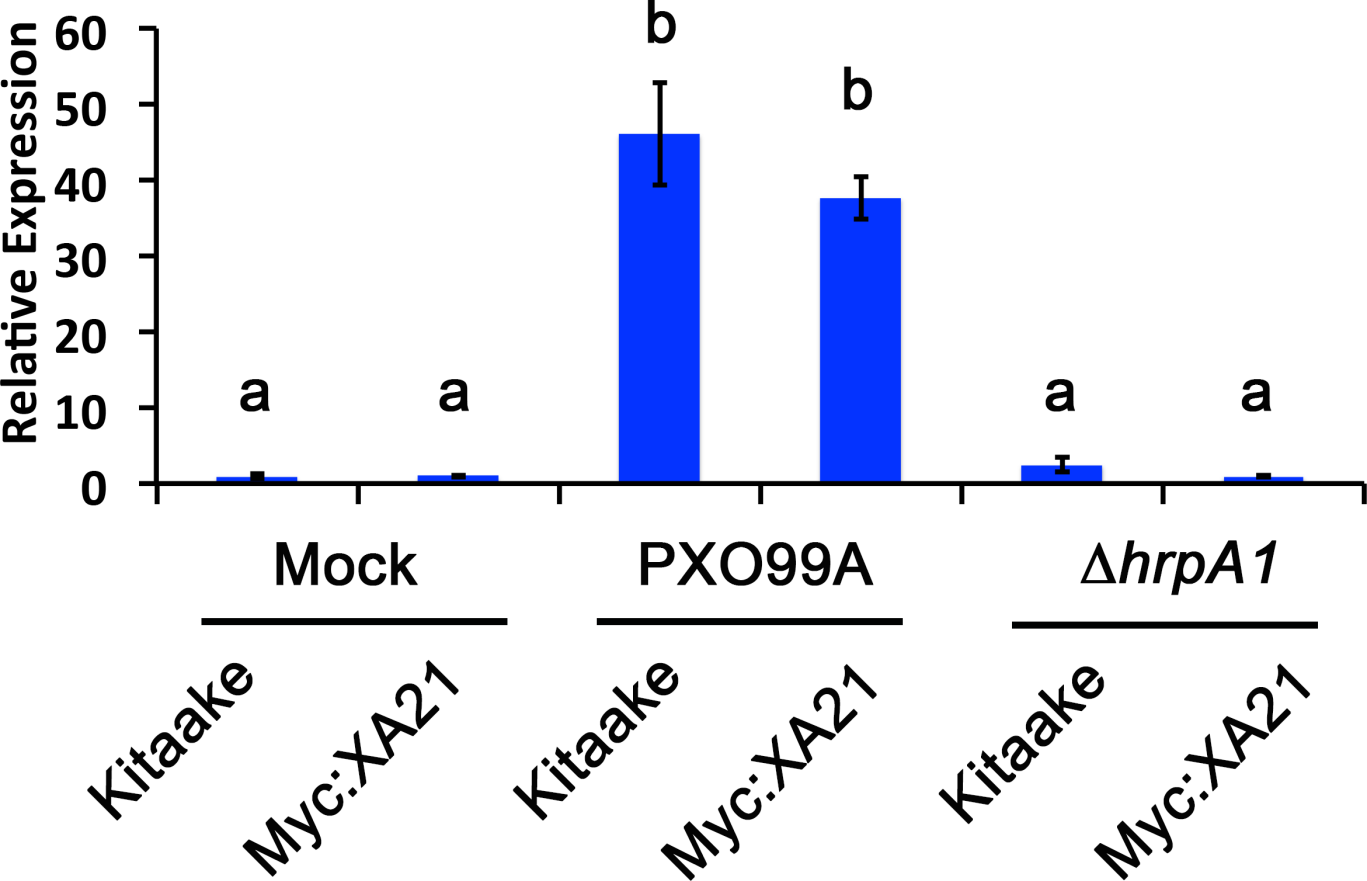
A marker gene of *Xanthomonas* infection, *Os8N3*, is up-regulated in PXO99A infected leaves. *Os8N3* expression in detached Kitaake and Myc:XA21 rice leaves with 10 mM MgCl_2_ mock treatment or infected with PXO99A or PXO99AΔ*hrpA1* (Δ*hrpA1*) at an O.D._600_ of 0.1. Letters represent statistically significant differences between mean expression values (*p* < 0.05) determined by using a Tukey–Kramer HSD test. This experiment was repeated three times with similar results.

We established a detached leaf infection assay to test if genes identified in the EFR:XA21:GFP experiments are representative of genes differentially regulated in *Xoo* infected Myc:XA21 rice. We observed bacterial ooze from the detached rice leaves three days after inoculation with *Xoo* strain PXO99A (Fig. 2). To further assess if *Xoo* infects rice leaves in our detached leaf infection assay, we measured the expression level of *Os8N3* (*LOC_Os08g42350*), which was previously shown to be up-regulated in rice upon *Xoo* infection and is thus a useful marker of successful infection (Yang, Sugio & White, 2006). For these experiments, we also included a mutant PXO99A strain (PXO99AΔ*hrpA1*) that is unable to infect rice as a control. The *hrpA1* gene encodes a pilus protein that is essential for type III-secretion of effectors required for host infection (Wengelnik et al., 1996). We observed that the PXO99AΔ*hrpA1 Xoo* mutant is unable to infect Kitaake and Myc:XA21 rice plants (Fig. S2). Both WT Kitaake and Myc:XA21 detached leaves express *Os8N3* at higher levels compared to mock treatments 24 hpi with WT PXO99A, but not with PXO99AΔ*hrpA1* (Fig. 3). These results demonstrate that *Xoo* infects detached rice leaves.

**Figure 4:**
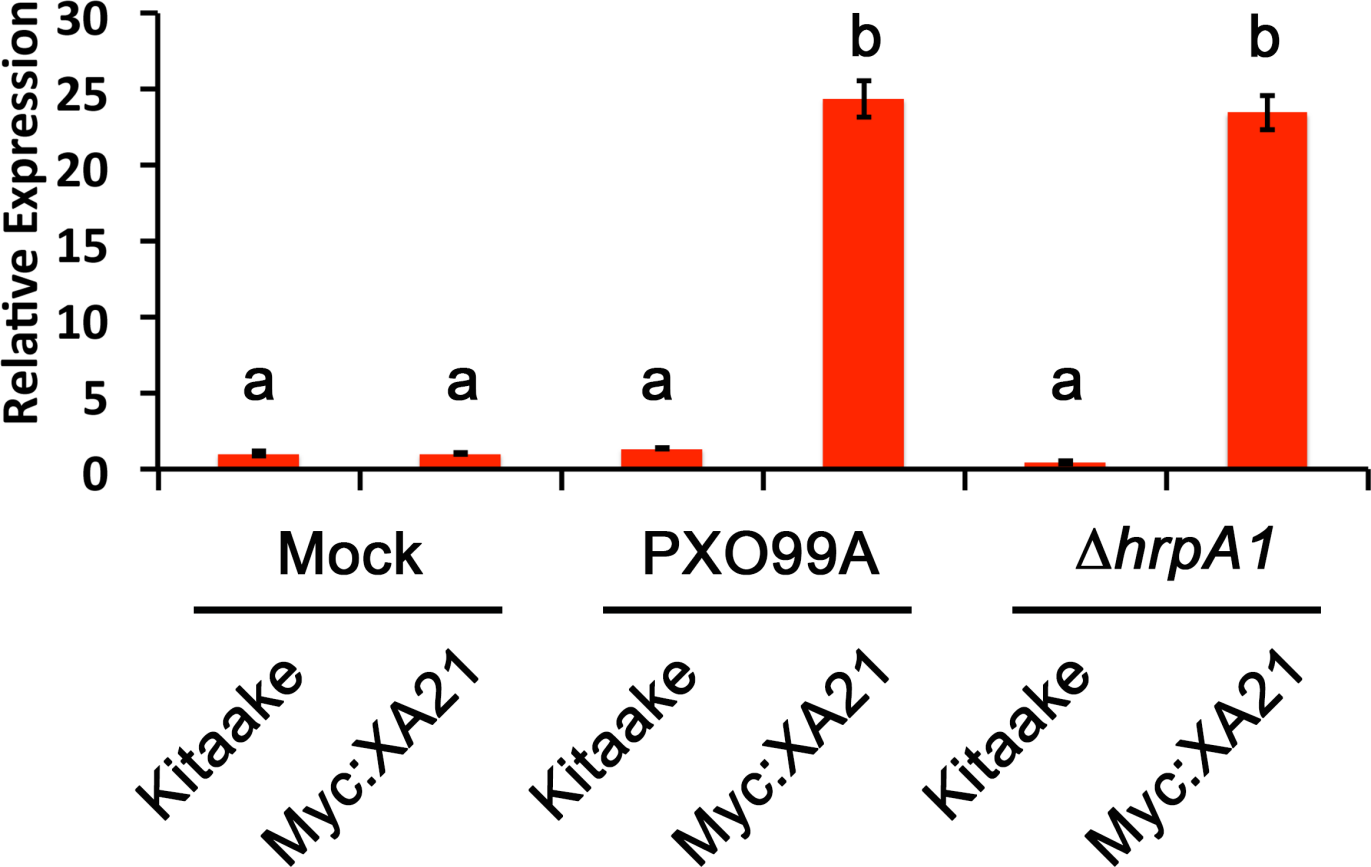
The stress-related marker gene *PR10b* is up-regulated in *Xanthomonas* infected XA21 rice. *PR10b* expression in detached Kitaake and Myc:XA21 rice leaves with 10 mM MgCl_2_ mock treatment or infected with PXO99A or PXO99AΔ*hrpA1* (Δ*hrpA1*) at an O.D._600_ of 0.1. Letters represent statistically significant differences between mean expression values (*p* < 0.05) determined by using a Tukey–Kramer HSD test. This experiment was repeated three times with similar results.

We next employed the detached leaf infection assay to examine the expression of the stress-related marker *PR10b* in *Xoo* infected Myc:XA21 rice leaves. Compared with mock treated controls, *PR10b* is up-regulated in flg22 treated rice, elf18 treated EFR:XA21:GFP rice and Myc:XA21 rice treated with the RaxX21-sY (Chen et al., 2014; Schwessinger et al., 2015a; Pruitt et al., 2015). Using qPCR, we detected significant up-regulation of *PR10b* expression in Myc:XA21 rice leaves 24 hpi with PXO99A and PXO99AΔ*hrpA1*. *PR10b* up-regulation was not observed in infected Kitaake leaves (Fig. 4). These results show that the detached leaf infection assay can be used to assess XA21-mediated marker gene expression and also indicate that RaxX expression or secretion is not affected by the Δ*hrpA1* mutation.

**Figure 5:**
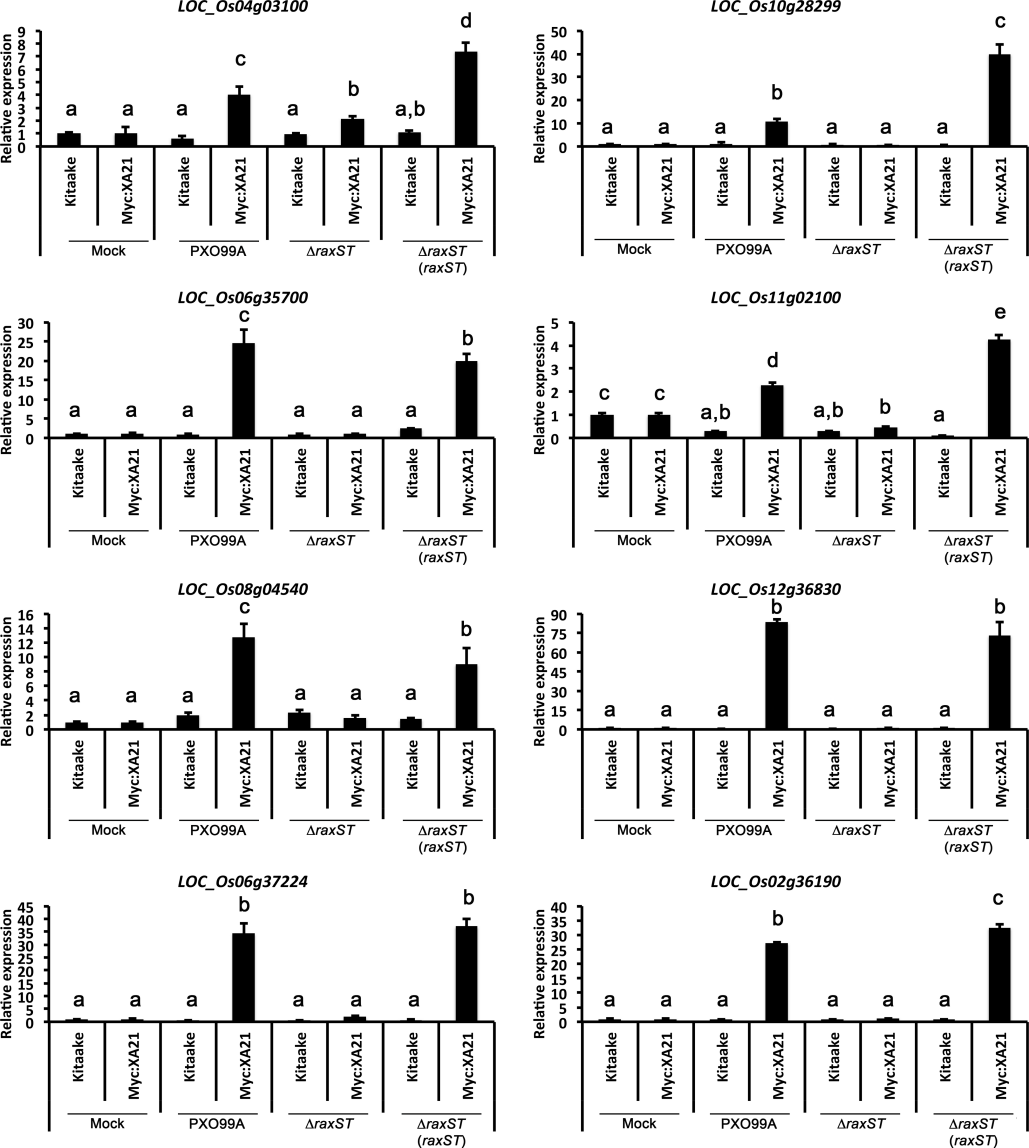
Eight marker genes are specifically up-regulated in detached rice leaves undergoing the XA21-mediated immune response. Expression of eight genes was measured in detached Kitaake and Myc:XA21 leaves infected with different *Xoo* strains. Mock samples were treated with 10 mM MgCl_2_. *Xoo* strains used for infection were WT PXO99A, a PXO99AΔ*raxST* mutant strain that evades XA21-mediated immunity (Δ*raxST*), and the PXO99AΔ*raxST* mutant strain complemented with *raxST* (Δ*raxST* (*raxST*)). Expression levels are normalized to *Actin* then compared to mock treated samples. Bars indicate mean expression levels ± standard deviation of three technical replicates. Letters represent statistically significant differences between mean expression values (*p* < 0.05) determined using a Tukey–Kramer HSD test. This experiment was repeated twice with similar results.

### Eight out of 11 genes induced in efl18 treated EFR:XA21:GFP rice are up-regulated in *Xoo* infected XA21 rice

We next employed the detached leaf infection assay to identify genes up-regulated upon *Xoo* infection of Myc:XA21 rice leaves. For these assays, we examined the gene expression of the 11 genes validated by qPCR analysis of elf18 treated EFR:XA21:GFP rice described above. We inoculated Kitaake and Myc:XA21 rice with WT PXO99A, PXO99AΔ*raxST* mutants, and PXO99AΔ*raxST* complemented with *raxST* (PXO99AΔ*raxST*(*raxST*)). *Xoo* strains carrying mutations in *raxST* do not activate XA21-mediated immunity (Da Silva et al., 2004; Pruitt et al., 2015). The expression of 8 of 11 genes was specifically up-regulated in detached Myc:XA21 rice leaves 24 hpi with PXO99A and PXO99AΔ*raxST* (*raxST*) but not in Myc:XA21 rice leaves infected with PXO99AΔ*raxST* (Fig. 5 and File S3). The 8 validated marker genes encode a putative subtilisin-like protein (*LOC_Os04g03100*), a reticuline oxidase-like protein precursor (*LOC_Os06g35700*), the decarboxylase OsTDC1 (*LOC_Os08g04540*) (Kang et al., 2007), a peroxidase precursor (*LOC_Os11g02100*), RsOsPR10a (*LOC_Os12g36830*) (Hashimoto et al., 2004; Takeuchi et al., 2011), CYP71Z7 (*LOC_Os02g36190*) (Li et al., 2013), OsKO5 (*LOC_Os06g37224*) (Itoh et al., 2004), and one protein without a putative function (*LOC_Os10g28299*). The 3 remaining genes that were up-regulated in elf18 EFR:XA21:GFP rice but not in Myc:XA21 rice leaves encode a isoflavone reductase (*LOC_Os01g13610*), a subtilisin-like protein (*LOC_Os04g03100*), and a reticuline oxidase-like protein precursor (*LOC_Os06g35700*).

## Discussion

In this study we identified 8 genes that are specifically up-regulated in both elf18 treated EFR:XA21:GFP and *Xoo* infected detached Myc:XA21 rice leaves. At the time of these experiments, the activator of XA21, RaxX, had not yet been identified (Pruitt et al., 2015). We therefore treated rice plants expressing the EFR:XA21:GFP chimera with elf18 to identify candidate marker genes because EFR:XA21:GFP rice are partially resistant to *Xoo* and respond to elf18 treatments as described above in the introduction. Our results show that even though the EFR:XA21:GFP-mediated response does not confer robust resistance to *Xoo* (Schwessinger et al., 2015a), similar genes are up-regulated during both EFR:XA21:GFP- and Myc:XA21-mediated responses (Fig. 5). Further studies are necessary to determine why the expression of EFR:XA21:GFP in rice does not confer robust resistance to *Xoo*.

We show that stress-related gene induction of *PR10b* in Myc:XA21 rice leaves is maintained in plants inoculated with PXO99AΔ*hrpA1* mutant strains. These results suggest that RaxX expression, modification and secretion is not compromised by the Δ*hrpA1* mutation. These results indicate that RaxX function is independent of type-III secretion mediated by *hrpA1*. It was previously reported that the *raxSTAB* operon, which encoded predicted components of a type-I secretion system, was required for the processing and secretion of the XA21 elicitor (Da Silva et al., 2004). Our finding that RaxX function is independent of *hrpA1*-mediated type-III secretion is consistent with the hypothesis that RaxX is a type I-secreted molecule (Da Silva et al., 2004; Pruitt et al., 2015) and may provide insight into the largely unknown biological function of RaxX.

The discovery of RaxX and the establishment of the detached leaf infection assay described here provide useful tools for studying XA21-mediated immunity. XA21 activation can be measured through ROS production and marker gene expression in detached leaves treated with the RaxX21-sY peptide (Pruitt et al., 2015; Schwessinger et al., 2015b). One advantage of this approach is that researchers can study XA21-mediated immunity without working with *Xoo*. Instead, researchers can activate XA21-mediated immunity by treating leaves with RaxX21-sY peptide rather than *Xoo*. This strategy eliminates the need for select agent permits, which are costly and time-consuming. The assay described in this study now allows researchers to use *Xoo* infected plants to monitor XA21 activation by gene expression, which was previously only possible using peptide treatments. This provides the benefit of monitoring bacterial induced genes, such as *Os8N3* (Fig. 3). While we are not able to definitively assess resistance versus susceptibility to *Xoo* using this assay, we demonstrate that we can use gene expression to monitor an immune response specifically mediated by XA21.

The detached leaf infection assay can also be used for other studies of bacterial-rice interactions. For example, this system can be used to study rice immune responses conferred by different resistance genes or induced by different bacterial strains. For example, the detached leaf infection assay can be used to study the immune response conferred by other rice *Xa* genes (Khan, Naeem & Iqbal, 2014) that confer resistance to *Xoo* such as *Xa3/Xa26*, which also encodes a cell surface receptor kinase (Xiang et al., 2006; Li et al., 2012). The detached leaf infection assay can also be adapted to study immune responses to other races of *Xoo* (Niño-Liu, Ronald & Bogdanove, 2006) or other *Xanthomonas* pathovars such as *Xanthomonas oryzae* pv. *oryzicola* (Raymundo, Perez & Co, 1992; Niño-Liu, Ronald & Bogdanove, 2006).

##  Supplemental Information

10.7717/peerj.2446/supp-1 Figure S1 Image of *Xoo* infection of detached rice leaves Image of detached rice leaf assay setup. 1.5-2cm detached leaves are floated on 1.5mL of bacterial suspension in 6-well flat bottom cell culture plates (approximately 12.5 × 8.5 × 2 cm).

10.7717/peerj.2446/supp-2 Figure S2 Infection with PXO99AΔ*hrpA1* mutants does not form lesions on Kitaake or XA21 rice leaves Kitaake or Myc:XA21 rice were inoculated with scissors dipped in PXO99A or PXO99AΔ*hrpA1* (Δ*hrpA1*) at an approximate cell density of 8x108 cells mL-1. Boxplots (red) represent distribution of lesion measurements from three different plants taken 14 days after infection with at least three measurements from each plant (*n* ≥ 9). Blue lines indicate standard deviation of the mean.

10.7717/peerj.2446/supp-3 Table S1 RNA sequencing sample treatment summary Table summarizes the experimental setup including the genotypes, time of treatment, and type of treatment used for samples used in RNA sequencing. There were three replicates for each sample for a total of 21 sequenced samples.

10.7717/peerj.2446/supp-4 File S2 Differentially regulated gene clade groups and GO term analysis

10.7717/peerj.2446/supp-5 File S1 Differentially regulated gene expression table with clade number and putatuve function

10.7717/peerj.2446/supp-6 File S3 Marker gene summary and primer information

## Additional Information and Declarations

### Data Availability

The following information was supplied regarding data availability: The National Center for Biotechnology Information Sequence Read Archive (SRA) BioProject ID PRJNA250865.
