# Supplementary figures and images for: XA21-specific induction of stress-related genes following Xanthomonas infection of detached rice leaves
Source: PeerJ. 2016 Sep 28;4:e2446. doi: 10.7717/peerj.2446 (PMC5045893; doi:10.7717/peerj.2446)

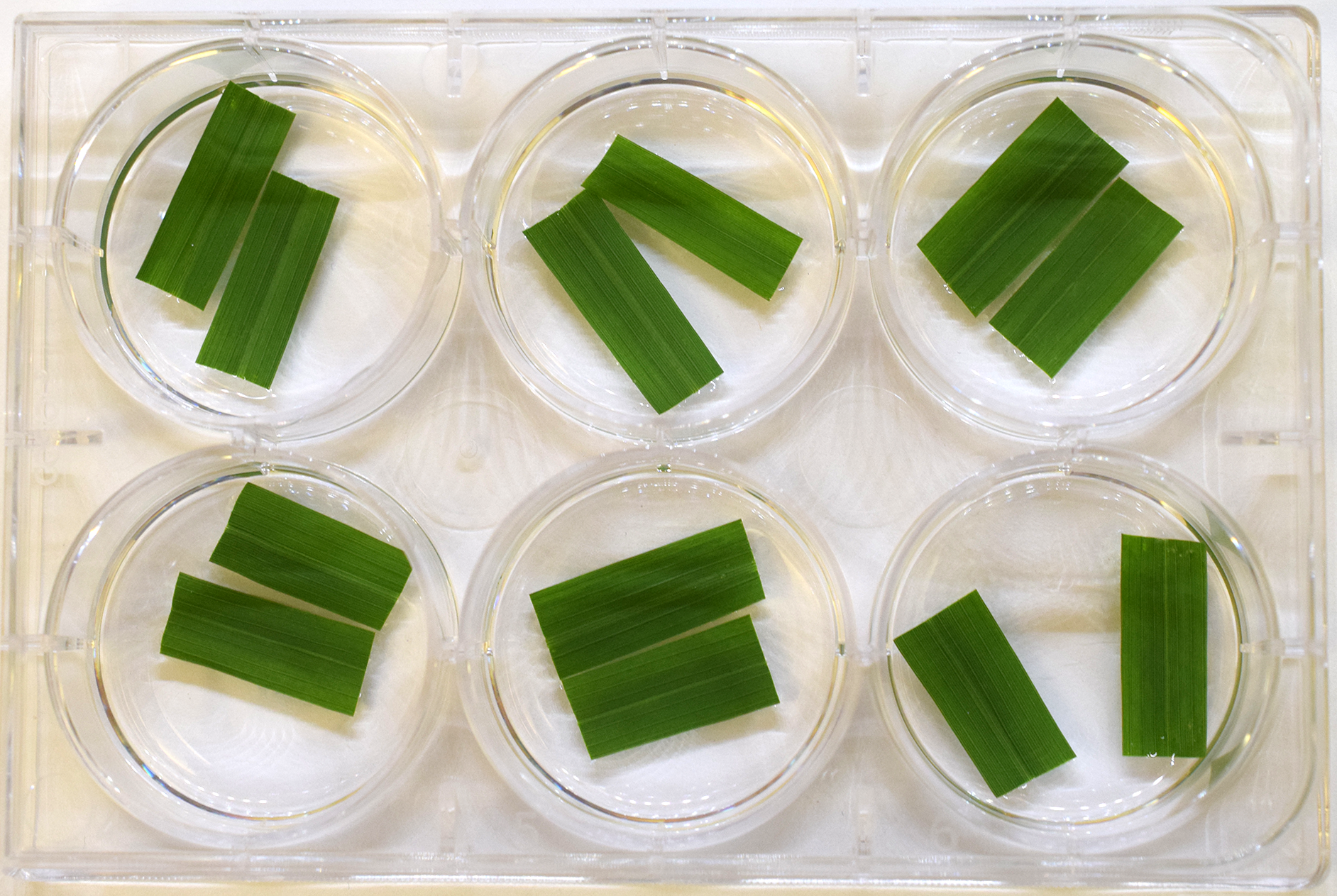

Supplement: Figure S1 — Image of detached rice leaf assay setup. 1.5-2cm detached leaves are floated on 1.5mL of bacterial suspension in 6-well flat bottom cell culture plates (approximately 12.5 × 8.5 × 2 cm). [file peerj-04-2446-s001.png]

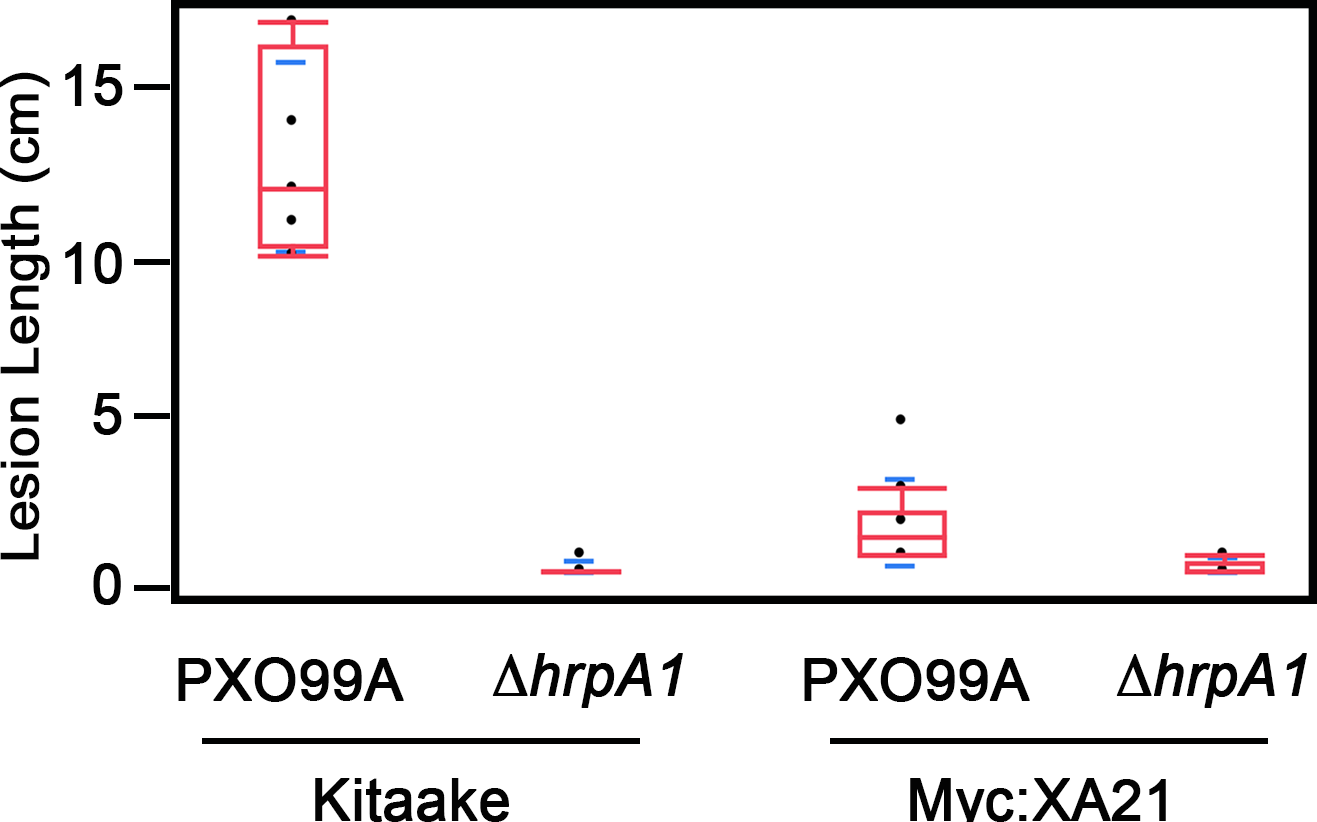

Supplement: Figure S2 — Kitaake or Myc:XA21 rice were inoculated with scissors dipped in PXO99A or PXO99AΔhrpA1 (ΔhrpA1) at an approximate cell density of 8x10^8^ cells mL-1. Boxplots (red) represent distribution of lesion measurements from three different plants taken 14 days after infection with at least three measurements from each plant (n ≥ 9). Blue lines indicate standard deviation of the mean. [file peerj-04-2446-s002.png]
